# Aloha Undergraduates: Development and Application of Local & Indigenous Topics into an Undergraduate Public Health Curriculum

**DOI:** 10.3389/fpubh.2019.00016

**Published:** 2019-02-12

**Authors:** Denise C. Nelson-Hurwitz, Lisa Kehl, Kathryn L. Braun

**Affiliations:** Office of Public Health Studies, University of Hawai‘i at Mānoa, Honolulu, HI, United States

**Keywords:** public health education, service learning, bachelors of public health, undergraduate education, indigenous health, curriculum development, high-impact educational practices, applied learning

## Abstract

As public health education expands to include undergraduate students, it is important to include discussion of local public health topics and issues to provide a sense of place to the educational experience. Inclusion of Native Hawaiian and indigenous issues and perspectives is also an established priority of the University of Hawai‘i system. To address both needs, a required course was created during development of a new Bachelor of Arts (BA) public health program at the University of Hawai‘i at Mānoa to specifically focus on discussion of local and indigenous public health topics of interest. Public Health Issues in Hawai‘i is an introductory course included early in the recommended undergraduate curriculum and emphasizes the application of public health skills and principles to local issues (e.g., state-level legislative awareness and local sustainability topics). The Public Health Issues in Hawai‘i course further challenges students to recognize public health practice in their daily activities, and encourages them to become actively engaged in local community issues early in their public health educational careers. Among multiple advantages, improved awareness of local health challenges and early connections to community members and organizations have been instrumental in actively engaging local students in their education, and has also proved beneficial for students participating in required undergraduate applied learning capstone experiences and entry-level public health careers following graduation. Here we present insights into course development, articulation with broader program curricula, and successes and challenges in the past 4 years of implementation and instruction.

## Background and Rationale

During development of the Bachelor of Arts in Public Health (BAPH) program at the University of Hawai‘i at Mānoa (UHM), faculty sought out opportunities for students to (1) connect and apply newly acquired public health content and skills to their individual communities, and (2) gain repetitive exposure to core public health content. There was a clear need for development of a basic introduction to public health course to provide foundational exposure to core skills and concepts, however, it was limited within a semester format. To help students apply public health principles to pressing local issues, a new course was developed—PH 202: Public Health Issues in Hawai‘i.

Throughout this course, students are able to apply core public health skills and concepts acquired during the prerequisite Introduction to Public Health course to local communities and gain awareness of local issues within a public health framework. In many cases, students are asked to actively engage in their local community, and participate in community events through service learning and community-based learning assignments. In-class, oral communication skills are consistently emphasized, as is the need for students to work collaboratively with peers on in-class activities and projects.

This class also serves as a key component of our introductory core series, and as a compliment to a required global health course (1). Following a broad introduction to public health, students are asked to use and apply public health content and skills in both a local context (PH 202: Public Health Issues in Hawai‘i) and a global context (PH 203: Introduction to Global Health), further providing opportunities for students to engage in issues of diversity and global learning. This dichotomy further allows students to clearly recognize the juxtaposition between application of public health in both a local and global health context.

The University of Hawai‘i system has identified itself as an indigenous-serving, Hawaiian place of knowledge^1^ PH 202 further serves as an opportunity to apply these intentions and address Native Hawaiian and indigenous health issues within the context of undergraduate public health education.

Students attending the University of Hawai‘i are predominantly Hawai‘i residents and frequently seek higher education with the intent of seeking employment within the state^1^. To best serve these students, it is helpful to support connections between students and local organizations relevant to their field of training, which may be future employers. Another key curricular component of the BAPH program at UHM is the capstone project requirement—the Applied Learning Experience (APLE) (2). Through this requirement, students engage in community internships or applied research experiences. Early exposure to local public health organizations through PH 202 also provides students with an awareness of sites with which to collaborate as relevant to their capstone projects.

## Pedagogical Framework

Multiple high-impact educational practices are applied throughout this course. As a highly diverse state with a large indigenous population and substantial minority and immigrant populations, diversity and global learning are of predominant importance throughout the course topics. Indigenous and immigrant health, culturally appropriate practices, and historical context of local public health issues are interwoven throughout the curriculum.

PH 202 also incorporates the critical component elements (CCEs) identified by the Association of Schools and Programs of Public Health (ASPPH)^2^, as well as Liberal Education and America's Promise (LEAP) learning objectives (3, 4). These objectives relate to inquiry and analysis, critical and creative thinking, written and oral communication, quantitative literacy, information literacy, teamwork, problem solving, social responsivity, and integrative learning.

To integrate and involve students actively in the local community, the course also capitalizes on service learning and community-based learning, both high impact educational practices (5). The clearest example of this is through the required ‘*aina* (land) connection experience, where students volunteer in environmental community workdays, then reflect on their experiences. Students also engage with current topics and issues in pairs or small groups through activities including community windshield assessments, public-health-in-the-news assignments, and community challenge/success presentations. Many of these assignments also apply collaborative learning practices, another high impact educational practice, as students work in small groups to design culturally tailored public service announcements and collaborate in legislative budget balancing activities. Collaborative learning also plays a key role in this course, as an early requirement in the BAPH curriculum. Team-based experiential learning is further supported in pedagological effectiveness when applied in higher education (6, 7), and has been specifically applied in undergraduate public health coursework (8). Through group activities, students often develop a cohort effect and relationships with peers that continue through their undergraduate experience.

### Developing the Course

Course development and design was led by formative research, including a literature review, web searches, best practices in pedagogical techniques, CEPH standards, key informant interviews with stakeholders, and review by departmental committees. Design and curriculum content were guided by recommendations from the literature (9–13) and the ASPPH Framing the Future Task Force^2^. The course was intended to build on topics and skills introduced during the prerequisite Introduction to Public Health course, allowing students a second exposure to key concepts in public health, as well as an opportunity to both observe and participate in local application of critical public health skills and concepts (14). Pedagogical approaches were incorporated to address variability among students in regards to learning style, with particular emphasis on application of experiential learning techniques and regular opportunities for student reflection.

Interviews were also conducted with key faculty specializing in Native Hawaiian and Indigenous Health, local community leaders, and university administration to ensure the developed course was culturally appropriate, incorporated an indigenous perspective, included strong local connections, and articulated well with existing curricular requirements and institutional learning objectives.

## Learning Environment

The University of Hawai‘i at Mānoa (UHM) is a public research university and the flagship campus for the University of Hawai‘i system. Student enrollment is 17,612 students, with undergraduate students comprising about 73% of enrollees^3^ Demographics reflect a student body comprised primarily of residents^3^ and high ethnic diversity (15). The University of Hawai‘i system has identified itself as an indigenous-serving institution and as a Hawaiian place of learning, clearly communicating the prioritization within the administration and across the multiple campuses.

Public Health Issues in Hawai‘i (PH 202) is a core, required course within the BAPH curriculum at UHM. It additionally serves as a component of our introductory core course series. The two additional courses of the series includes Introduction to Public Health (PH 201) as a prerequisite, and Introduction to Global Health (PH 203) as a course taken concurrently with, or following completion of, PH 202. Three public health faculty with experience in undergraduate instruction teach each of the three introductory public health courses independently. Logistically, PH 202 is taught in two class sessions of 75 min each per week, and offered during the fall and spring semesters, with additional offerings in the summer dependent on instructor availability. Since inception, over 300 students have enrolled in PH 202, and class size ranges from 22 to 42 students per semester.

## Learning Objectives

The course has 12 learning objectives: (1) Develop a respect for culture and place in Hawai‘i; (2) Understand health disparities caused by historical and cultural trauma among indigenous peoples, particularly those of Pacific Island descent; (3) Discuss the implications of culture on health and identify the need for culturally sensitive care; (4) Connect the need for sustainability and the importance of environmental health to human health; (5) Develop connections between students and local communities, and encourage student participation as engaged citizens; (6) Identify local community strengths and resources; (7) Promote self-reflection to identify and recognize personal skills and how they may be applied to contribute to local communities; (8) Develop an understanding of the legislative process, and an appreciation for the complexities of that process; (9) Translate social justice issues and health priorities into actionable steps; (10) Empower students to engage in health policy and advocacy; (11) Develop confidence in oral communication and public speaking; and (12) Develop skills in teamwork and collaboration among peers.

## Pedagogical Format

Throughout this course, students are involved in applying core public health skills and concepts to the local community, while improving awareness of local issues within a public health framework. To achieve this, as well as to meet the learning objectives above, several key assignments serve to anchor the course.

### “Public Health in Action” Photovoice Assignment

For the first assignment of the semester, students are asked to explore their community and identify three observations of “public health in action.” They take pictures of their observations, upload them into Google Slides, and caption the images to explain their observations. In class, each student reports back to the class using their Google Slides presentation and explaining their findings. This is followed by a class discussion about both similar and unique observations among students. The objective of this assignment/activity is to get the students to begin to explore their community, and to get them to realize that public health is everywhere.

### Public Health in the News Presentations

In an effort to increase student awareness of local, contemporaneous public health issues across the state, students are asked to use local media sources to complete a brief worksheet. The students then present their stories in class as a panel guest hosting the fictitious, local evening news. This activity additionally serves as an opportunity for students to engage with university library resources, and practice accessing high quality local media sources for use in an academic setting.

### ‘*Aina* (Land) Connection Experience

In an effort to promote experiential learning outside of the classroom, to encourage students to engage in local community efforts, and to promote a community-grounded understanding of Hawaiian values and culture, students are required to participate in one ‘*aina* (Land) Connection Experience at some point during the semester. Approved ‘*aina* (land) connection experiences include a session (generally 2–4 h) of volunteer work at the University of Hawai‘i at Mānoa's *lo‘i* (taro patch), located adjacent to campus, or engagement in environmental community workdays. Past events have also included organized beach clean ups and dedicated workdays at community gardens/farms or *i‘a loko i‘a* (indigenous fish ponds). Students have the opportunity to request that similar, self-identified experiences be counted with instructor approval. As evidence of participation, students are asked to provide a photo of themselves taken at the site during the activity, and to write a brief reflection of their experience.

Culturally, Native Hawaiians and other Pacific Islanders feel strong connections to their natural environment (16). By encouraging students to actively engage in their local communities, to work alongside their neighbors, and to participate in service activities centered on sustainability, students are able to develop a deeper understanding of the Pacific culture and stronger appreciation for public health topics of environmental health and community engagement.

### Community Windshield Assessment

For this assignment, students are once again asked to explore their community. This time they use observation and assessment to complete a worksheet about the health and safety of their community in regard to food availability, opportunities for physical activity, sidewalk existence and conditions, health care facilities, and overall built environment. The objective of this assignment is for students to see their community through the lenses of public health and built environment by identifying elements that facilitate and hinder health within their community.

### Challenge/Success Presentations

Public Health Challenge and Success Presentations are assigned in lieu of a final exam. For this assignment, students work in assigned pairs to develop and deliver an 8- to 10-min presentation regarding a public health challenge and a public health success in Hawai‘i. The presentations must include the following elements for the challenge and the success: (1) brief background; (2) description; (3) key stakeholders and interests; and (4) public health significance. For the challenge, students also present potential solutions, and for the success they also speculate how the public health community beyond Hawai‘i can learn from it. A brief 2–3 min question and answer session follows each pair presentation. This assignment requires students to identify strengths and challenges of local public health and public health issues, and also allows for assessment of student oral presentation skills early in their public health degree program. Past selected challenges in local public health have included issues such as homelessness and invasive miconia plants, which prevents the growth of native plants and increases the risk of erosion with its shallow root systems. Past successes have included implementation of smoking bans on local public beaches, and increased nutritional access through acceptance of EBT cards at local farmers markets.

### Weekly Blog Reflections

Students are required to write weekly blogs/reflections and post them on the course website. Each blog is a critical synthesis of that week's topics, discussions, and activities written as student responses to reflection questions (see Table 1). Additionally, students are encouraged to respond to other student's blog entries to promote group discussion and learning. For the course instructor, weekly blogs also provide an opportunity to assess student mastery of content and skill development (17–19).

**Table 1:** Sample blog prompts.

**Topic**	**Blog prompt**
Public health in action	Please share what your experience was like taking photos for your Public Health in Action assignment. How did you decide what you were going to take pictures of? What challenges did you encounter? What would you do differently if you had to do it again? What did you learn from the assignment?
Native Hawaiian health and cultural practices	In relation to the class discussion we had on Monday about cultural practices among Native Hawaiians/Indigenous People that may improve health, I would like you to come up with a strength/activity from within a culture you identify with that could be beneficial to health, why you do or don't take advantage of this strength/activity, and if it benefits your health. (Remember culture doesn't just mean ethnicity. A culture can mean student culture or local culture, etc.)
Health policy & legislative process	For this week, I would like you to find a bill on a topic that interests you. Write what the bill is about (include bill number) and why it is important to you. Discuss the public health issue the bill addresses and how the bill is a possible solution to that public health issue. Also, find out who your State Senator and Representative are and what committees they sit on. Then discuss how they could be useful in passing the bill you selected.
Built environment	Please talk about your group process for creating your ideal community. How did you work together? What role did you take on? How did you contribute to the process? After hearing other groups' presentations and reflecting on the activity, what else would you include in your community that you left our originally and justify why you think it is important.
Local department of health and organizations	Find and describe one program or branch at the Hawai‘i Department of Health that you may want to work for and why?

## Outcomes and Assessment Results

Rubrics are used to assess mastery of assignments and learning objectives, and these data suggest that >88% of students leave the course with solid mastery (either excellent or good), of the learning objectives (see Table 2).

**Table 2:** Mastery of learning objectives as assessed by the instructor through completion of assignments—most current semester (Spring 2018).

**Learning Objectives**	**Activities/assignments to demonstrate mastery**	**Excellent**	**Good**	**Poor**
1) Develop a respect for culture and place in Hawai‘i	‘*Aina* (Land) connection experience	19 (86%)	2 (9%)	1 (5%)
2) Understand health disparities caused by historical and cultural trauma among indigenous peoples, particularly those of Pacific Island descent	Discussions with native Hawaiian guest speakers & videos/ discussion of compact of free-association (COFA) migrants	21 (95%)	0	1 (5%)
3) Discuss the implications of culture on health and identify the need for culturally sensitive care	Developing a culturally appropriate public service announcement	18 (82%)	0	4 (18%)
4) Connect the need for sustainability and the importance of environmental health to human health	‘*Aina* (Land) connection experience	19 (86%)	2 (9%)	1 (5%)
	Developing a group built environment	17 (77%)	0	5 (23%)
5) Develop connections between students and local communities, and encourage student participation as engaged citizens	‘*Aina* (Land) connection experience	19 (86%)	2 (9%)	1 (5%)
	Community windshield assessment	22 (100%)	0	0
6) Identify local community strengths and resources	Challenge/success presentations	9 (41%)	12 (54%)	1 (4%)
7) Promote self-reflection to identify and recognize personal skills and how they may be applied to contribute to local communities	Weekly blog reflections	19 (86%)	3 (14%)	0
8) Develop an understanding of the legislative process, and an appreciation for the complexities of that process	Balancing the state budget activity & debate	18 (82%)	0	4 (18%)
9) Translate social justice issues and health priorities into actionable steps	Balancing the state budget activity & debate	18 (82%)	0	4 (18%)
10) Empower students to engage in health policy and advocacy	Discussions with community policy advocates & exercise in writing legislative testimony	10 (45%)	5 (23%)	7 (32%)
11) Develop confidence in oral communication and public speaking.	“Public health in action” photovoice assignment	19 (86%)	3 (14%)	0
	Developing a group built environment	17 (77%)	0	5 (23%)
	Challenge/success presentations	9 (41%)	12 (54%)	1 (4%)
12) Develop skills in teamwork and collaboration among peers.	Developing a culturally appropriate public service announcement	18 (82%)	0	4 (18%)
	Developing a group built environment	17 (77%)	0	5 (23%)
	Challenge/success presentations	9 (41%)	12 (54%)	1 (4%)

Data from course evaluations and exit questionnaires additionally suggest that the students appreciate the approach of the course and the challenge to apply concepts from introductory public health to the local level through this subsequent course. In global appraisal, 97–100% of students rate the course overall as “very good” or “good” on a 5-point likert scale in end-of-semester evaluations (Spring 2016, Fall 2017).

Student feedback from end-of-semester evaluations and periodic mid-semester assessments, are used to refine course materials and improve articulation of the course with the broader undergraduate public health curriculum. Informal assessment is also conducted during grading and review of weekly blog reflections, where students express concrete and actionable responses to prompts and demonstrate competence in the course material. Anecdotal evidence from instructors in subsequent courses suggest students are mastering course content and are able to apply developed skills in later coursework.

## Discussion

Since initial development, implementation has been challenged by the need to scale the course to accommodate a larger class size and concerns about overburdening community guest speakers. Early course sizes (roughly 12–20 students per semester) accommodated seminar-style discussions and activities. However, as the BAPH program rapidly expanded, there was a need to increase the capacity of the course to accommodate 30–40 students per semester. To address this need, activities were scaled back slightly in scope, and some activities initially completed in pairs are now completed by groups of 3–5 students. Application of technological support further assisted in expansion. Course management software helped to track class attendance, and was later used to administer short quizzes on assigned readings and videos to ensure assignments were read and videos viewed prior to class. Google Slides was later utilized to minimize transition time between challenge/success presentations, as students were required to share their completed slides with the instructor prior to class and upload them to the shared drive.

The PH 202 class has been consistently taught two to three times per year for the last 4 years. Though the topics and curriculum have remained consistent, the regularity of the course has led to a general rotation of local guest speakers. For example, a guest lecture focused on local legislative advocacy has featured, in rotation, representatives from the Hawai‘i Public Health Institute, the Hawai‘i-Pacific affiliate of the American Cancer Society Cancer Action Network, and a local non-profit organization, Healthy Mothers, Healthy Babies. This practice ensures local community agencies (particularly grassroots organizations with limited staff) are not overtaxed in their donation of time and also keeps the course fresh each semester as new perspectives are added.

Through implementation of this course, students demonstrate an improved awareness of local health challenges and actively engage in local community practices. The course helps students to connect newly acquired public health content and skills to issues many have seen first-hand in their home communities. Developing connections with local public health organizations and practitioners also help students to identify potential sites for required capstone experiences and future employment. Course implementation further fosters collaboration with local public health organizations and practitioners, improving community awareness of our program and students.

Public health undergraduate programs may benefit from development of coursework reflecting local and indigenous public health topics, or from the integration and application of community engagement into existing coursework. To begin development of such a course, institutions may consider outreach to local public health organizations (e.g., non-profit organizations), indigenous health-serving institutions, and the local department of health. Current public health faculty with local community connections (e.g., grant-related collaborations), may be helpful in facilitating development of these early relationships. Discussion with local organizations may support in identification of public health challenges most relevant to the local community, which may then be incorporated into the newly developed course.

## Ethics Statement

This study was carried out in accordance with the recommendations of the University of Hawai‘i (UH) Human Studies Program as exempt from federal regulations pertaining to the protection of human research participants. Authority for the exemption applicable is documented in the Code of Federal Regulations at 45 CFR 46.101(b) 4. The protocol was approved by the Office of Research Compliance, University of Hawai‘i system (Protocol Number 2018-00751).

## Author Contributions

DN-H and LK contributed conception and design of the course and all associated assignments and activities. DN-H wrote the first draft of the manuscript. All authors wrote sections of the manuscript. All authors contributed to manuscript revision, read, and approved the submitted version.

### Conflict of Interest Statement

The authors declare that the research was conducted in the absence of any commercial or financial relationships that could be construed as a potential conflict of interest.

## Footnotes

^1^http://manoa.hawaii.edu/about

^2^http://www.aspph.org/educate/framing-the-future/

^3^https://manoa.hawaii.edu/about/
